# Editorial: Unraveling the long-term effects of COVID-19

**DOI:** 10.3389/fncel.2026.1926660

**Published:** 2026-08-13

**Authors:** Romina Vuono, Kieren S. J. Allinson, Donato Zipeto, Nigel Temperton

**Affiliations:** 1 Medway School of Pharmacy, Universities of Greenwich and Kent, Chatham, United Kingdom; 2 Department of Clinical Neurosciences, University of Cambridge, Cambridge, United Kingdom; 3 Department of Neurosciences, Biomedicine and Movement Sciences, University of Verona, Verona, Italy

**Keywords:** SARS-CoV-2, COVID-19, long COVID, chronic inflammation, neurological symptoms, neurodegenerative diseases, cancer, artificial intelligence

More than 6 years after the emergence of SARS-CoV-2, the scientific and medical communities continue to confront one of the pandemic's most complex challenges: persistent symptoms and new health complications after resolution of the acute infection. Commonly referred to as long COVID or post-acute sequelae of SARS-CoV-2 infection (PASC), this condition has evolved from an early clinical observation into a major health concern affecting millions of individuals worldwide. Although substantial progress has been made in understanding the acute manifestations of COVID-19, the mechanisms underlying persistent symptoms remain incompletely defined, creating challenges for diagnosis, treatment, and long-term care.

**Figure 1:**
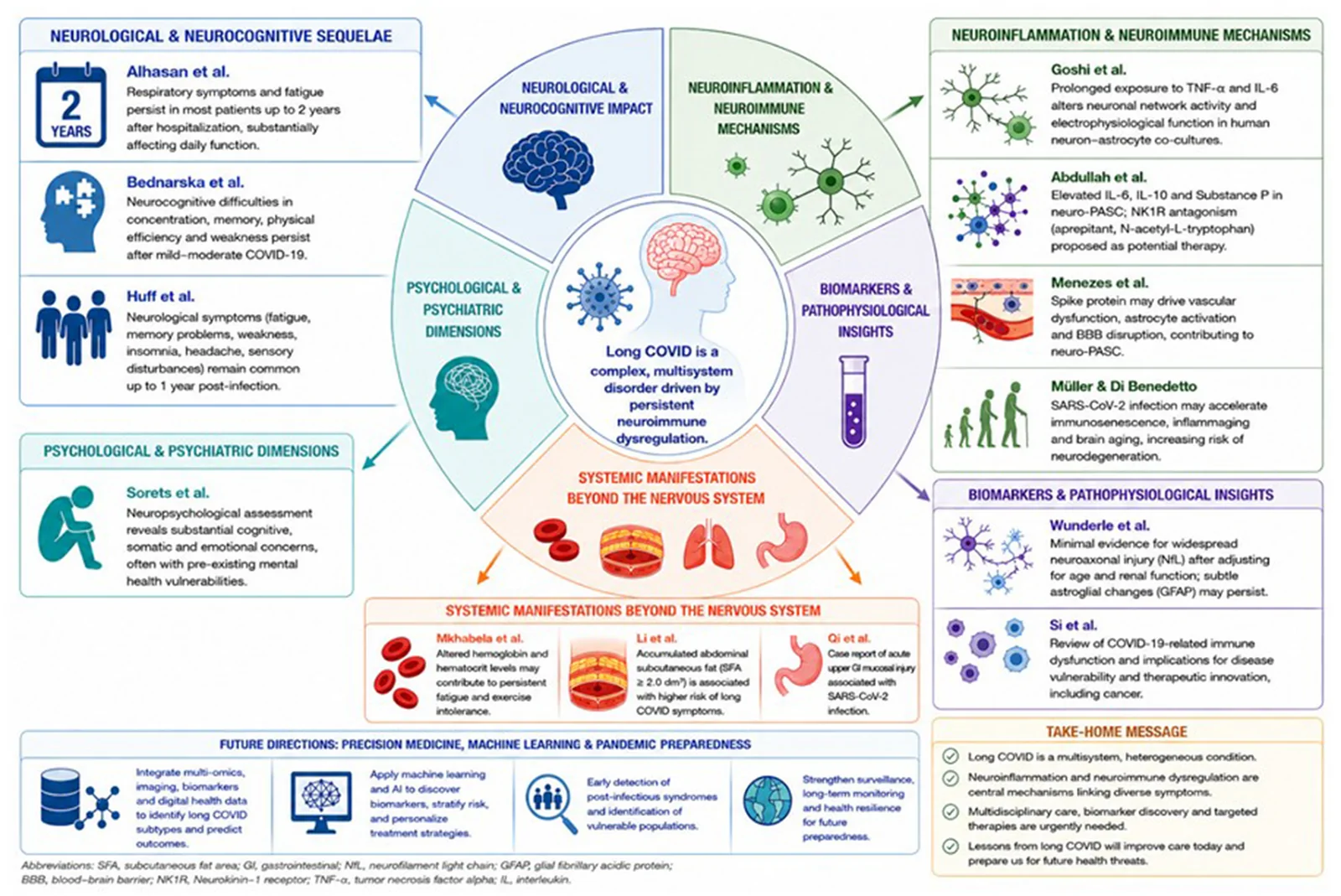
Overview of the key findings from the 13 contributions to the Research Topic *Unraveling the long-term effects of COVID-19*.

The contributions assembled in this Research Topic provide a comprehensive and multidisciplinary examination of the broad systemic consequences of SARS-CoV-2 infection (Figure 1). Collectively, they highlight the complexity and heterogeneity of long COVID while advancing understanding of its mechanisms and potential therapeutic targets. Patients may experience persistent symptoms across the nervous, respiratory, cardiovascular, gastrointestinal, endocrine, and immune systems, often with fluctuating severity and unpredictable clinical courses. Among these manifestations, neurological and neuropsychiatric symptoms including cognitive impairment, fatigue, memory deficits, sleep disturbance, anxiety, depression, and altered sensory perception, remain among the most frequently reported and disabling. Several studies in this Research Topic support neuroimmune dysregulation as a key driver of persistent neurological symptoms.

Abdullah et al. identified elevated circulating levels of IL-6, IL-10, and Substance P in individuals with long COVID-associated neuropsychological symptoms, reinforcing the role of persistent immune activation and neuroinflammatory signaling in the pathogenesis of neuro-PASC. Computational analyses further identified Neurokinin-1 receptor antagonism as a potential therapeutic strategy, with aprepitant and N-acetyl-L-tryptophan emerging as promising candidates for future investigation. Their study suggests that dysregulated inflammatory pathways may be clinically actionable targets.

Goshi et al. extended these mechanistic insights by examining prolonged inflammatory exposure in human neuron-astrocyte co-cultures. Chronic exposure to TNF-α and

IL-6 significantly altered neuronal network activity, even without direct viral infection. These findings indicate that sustained inflammatory signaling alone can disrupt neuronal function and connectivity, providing a biologically plausible explanation for the cognitive dysfunction and “brain fog” after viral clearance.

Complementing these cellular studies, Menezes et al. proposed a comprehensive hypothesis linking SARS-CoV-2 spike protein persistence to the development of neuro-PASC. Their review integrated evidence of blood–brain barrier disruption, endothelial injury, vascular dysfunction, astrocyte activation, and pericyte-mediated pathology as interconnected contributors to chronic neurological symptoms. This offers important insights into how persistent viral components may sustain neuroimmune activation and tissue dysfunction long after an acute infection.

The broader implications of these neuroimmune processes were explored by Müller and Di Benedetto who examined the relationship between COVID-19, immunosenescence, inflammaging, and brain aging. They proposed that SARS-CoV-2 infection may not only trigger transient inflammatory responses but also accelerate aging-associated neuroimmune pathways, potentially increasing susceptibility to cognitive decline and neurodegenerative diseases. This intersection between long COVID and biological aging warrants further study, particularly because of its public health implications.

Clinical studies reinforce the relevance of these mechanistic observations and show that long-term neurological sequelae are not limited to individuals with severe acute disease symptoms. Bednarska et al. reported persistent neurocognitive abnormalities after mild-to-moderate COVID-19, including impaired concentration, memory difficulties, generalized weakness, and reduced physical performance. These symptoms were common among non-hospitalized individuals, challenging the assumption that long-term outcomes are determined mainly by acute disease severity and underscoring the need for broader surveillance and follow-up strategies.

Similarly, Huff et al. provided longitudinal data from Peru, showing that neurological symptoms may persist for up to 1 year after infection. Fatigue, weakness, memory impairment, irritability, insomnia, and musculoskeletal complaints remained prevalent, illustrating the chronic, fluctuating nature of Neuro-PASC and underscoring the global relevance of long COVID.

While evidence for persistent neuroimmune dysfunction continues to grow, not all studies support the presence of ongoing structural brain injury. Wunderle et al. assessed circulating biomarkers of neuronal and astroglial damage, specifically neurofilament light chain (NfL) and glial fibrillary acidic protein (GFAP), in individuals with long COVID. After adjustment for age and renal function, evidence of persistent neuroaxonal injury was minimal. A modest increase in GFAP suggested subtle astroglial involvement, but widespread neuronal damage was not observed. These findings are particularly important because they help refine current models of long COVID by distinguishing chronic neuroinflammation from progressive neurodegeneration, which is critical for developing accurate biomarkers and targeted therapeutic interventions.

The psychosocial dimensions of long COVID also received significant interest. Sorets et al. found that many patients referred for neuropsychological assessment reported substantial cognitive, somatic, and emotional concerns, often alongside pre-existing mental health vulnerabilities. Their findings highlight the importance of considering psychiatric history, trauma exposure, and psychological resilience in evaluating long COVID. Biological and psychosocial factors should be viewed as interconnected contributors to the patient's outcomes, making integrated neurological, psychiatric, and rehabilitative approaches essential.

Although neurological manifestations are a defining feature of long COVID, several studies emphasize that the condition is fundamentally multisystemic. Alhasan et al. reported that respiratory symptoms and fatigue remained highly prevalent even 2 years after hospitalization and substantially affected functional status and daily activities, regardless of age, sex, or initial disease severity. These findings show that long COVID can impose a substantial long-term burden on quality of life and require sustained long-term clinical monitoring.

The physiological basis of persistent fatigue and exercise intolerance remains under investigation. Mkhabela et al. examined hemoglobin and haematocrit levels among adults with prior COVID-19 to assess possible effects on oxygen-carrying capacity. Although major abnormalities were not consistently observed, the study highlights the need to consider hematological nutritional, metabolic, and systemic factors when evaluating recovery trajectories.

Similarly, Li et al. demonstrated that increased abdominal subcutaneous adiposity was associated with a greater likelihood of developing persistent symptoms among non-hospitalized patients. These findings extend prior links between obesity and adverse acute COVID-19 outcomes and suggest that body composition and metabolic status may continue to influence recovery. They further support the critical role of host factors in shaping the risk and clinical presentation of long COVID.

COVID-19-associated pathology can also involve organ systems that have received less attention. Qi et al. presented a case of acute upper gastrointestinal mucosal injury associated with SARS-CoV-2 infection, providing direct evidence of gastrointestinal involvement through ACE2-associated mechanisms and illustrating the broad tissue tropism of the virus.

Finally, the long-term implications of COVID-19 may extend beyond the individuals with persistent symptoms. Si et al. discussed how post-COVID immune dysregulation, including T-cell exhaustion and altered inflammatory responses, may influence cancer biology and therapeutic strategies in non-small cell lung cancer. Their review suggests that SARS-CoV-2 infection may reshape disease susceptibility, progression, and treatment responses in chronic conditions beyond the immediate post-infectious period.

Taken together, the articles in this Research Topic support several conclusions. Long COVID is a complex multisystem disorder characterized by persistent neurological, immunological, psychological, and physiological abnormalities. Chronic inflammation and neuroimmune dysregulation are central mechanistic themes linking diverse clinical manifestations. Substantial heterogeneity among affected individuals indicates that multiple biological pathways may contribute to symptoms persistence. Effective management will, therefore, require multidisciplinary approaches that integrate biomarker discovery, mechanistic research, rehabilitation, mental health support, and targeted therapies.

Looking ahead, advances in machine learning and artificial intelligence offer unprecedented opportunities to integrate clinical, molecular, imaging, and digital health datasets to identify long COVID subtypes, predict outcomes, discover biomarkers, and personalize treatment. These approaches may also strengthen future pandemic preparedness by enabling earlier detection of post-infectious syndromes, identification of vulnerable populations, and real-time monitoring of long-term health consequences after emerging infectious diseases. Lessons from long COVID will improve care for current patients and help shape more resilient and responsive healthcare systems for future global health emergencies.

